# Improved prostate cancer diagnosis using a modified ResNet50-based deep learning architecture

**DOI:** 10.1186/s12911-024-02419-0

**Published:** 2024-01-24

**Authors:** Fatma M. Talaat, Shaker El-Sappagh, Khaled Alnowaiser, Esraa Hassan

**Affiliations:** 1 Faculty of Artificial Intelligence, Kafrelsheikh University, Kafrelsheikh, 33516 Egypt; 2 Faculty of Computer Science & Engineering, New Mansoura University, Gamasa, 35712 Egypt; 3 Faculty of Computer Science and Engineering, Galala University, Suez, 435611 Egypt; 4 Information Systems Department, Faculty of Computers and Artificial Intelligence, Benha University, Banha, 13518 Egypt; 5 College of Computer Engineering and Sciences, Prince Sattam Bin Abdulaziz University, Al Kharj, 11942 Saudi Arabia

**Keywords:** Prostate cancer detection, Convolution neural network, ResNet50, Dual optimizer

## Abstract

Prostate cancer, the most common cancer in men, is influenced by age, family history, genetics, and lifestyle factors. Early detection of prostate cancer using screening methods improves outcomes, but the balance between overdiagnosis and early detection remains debated. Using Deep Learning (DL) algorithms for prostate cancer detection offers a promising solution for accurate and efficient diagnosis, particularly in cases where prostate imaging is challenging. In this paper, we propose a Prostate Cancer Detection Model (PCDM) model for the automatic diagnosis of prostate cancer. It proves its clinical applicability to aid in the early detection and management of prostate cancer in real-world healthcare environments. The PCDM model is a modified ResNet50-based architecture that integrates faster R-CNN and dual optimizers to improve the performance of the detection process. The model is trained on a large dataset of annotated medical images, and the experimental results show that the proposed model outperforms both ResNet50 and VGG19 architectures. Specifically, the proposed model achieves high sensitivity, specificity, precision, and accuracy rates of 97.40%, 97.09%, 97.56%, and 95.24%, respectively.

## Introduction

Prostate cancer is one of the common types of cancer in men, and it is estimated that 1 out of 9 men will be diagnosed with prostate cancer at some point during their lifetime [1–3]. Prostate cancer can often be treated successfully if it is detected early, so it is important for men to get regular screenings to check for any signs or symptoms [4–8]. AI techniques are being used to detect prostate cancer to improve accuracy and reduce costs, such as Machine Learning (ML) and Deep Learning (DL), which are used to analyze MRI scans and CT scans to analyze patient data such as age, race, family history, and lifestyle factors. The use of DL for prostate cancer detection can help reduce costs by reducing the need for expensive biopsies and other tests. It can also help improve accuracy by providing more accurate results than traditional methods [9]. However, there are some challenges associated with using AI for prostate cancer detection. AI algorithms cannot accurately distinguish between benign and malignant tumors due to their complexity.

DL has the potential to revolutionize prostate cancer detection and provide more accurate results than traditional methods [10].

In this paper, we present a Prostate Cancer Detection Model (PCDM) depends on a modified ReseNet, a faster R-CNN mask, and dual optimizers (Adam and SGD) for detecting prostate cancer that applied on Prostate Cancer dataset [11–14]. PCDM model combines the power of DL with the accuracy of traditional methods to provide an effective method for detecting prostate cancer [15, 16].

The modified ReseNet model is used to extract features from the images, while the Faster R-CNN model is used to classify them. The dual optimizers (Adam and SGD) are used to optimize the parameters of the models, ensuring that they can accurately detect prostate cancer. The results of this technique have been impressive [12, 17]. It has been shown to be more accurate than traditional methods in detecting prostate cancer, with a sensitivity of up to 95%. Furthermore, it has been shown to be faster than traditional methods, taking only a few minutes for each image for quickly and accurately detecting prostate cancer in patients. The main contributions of this paper are as follows:

1. We propose a deep learning model based PCDM based on MRI images to accurately detect prostate cancer. The new architecture advances the current DL literature by proposing a modified version of the ResNet architecture.
2. The proposed PCDM uses ReseNet to effectively handle complex features, which can be crucial in detecting cancerous cells and achieving high accuracy in detecting prostate cancer cells.
3. The proposed study uses two different optimizers, Adam, and stochastic gradient descent (SGD), to train the PCDM to achieve a better balance between accuracy and efficiency in the training process.
4. The resulting model can help in the early detection of the disease. The PCDM has the potential to be applied to other medical imaging tasks beyond prostate cancer detection.

The structure of the paper is organized as follows. Literature review Section describes the literature review and deep learning work to recognize prostate cancer lesions. The suggested system and the representation and description of the dataset are found in Prostate cancer detection technique Section . Implementation and evaluation Section presents the experiential results. Discussion and conclusion Section concludes the main points of this work as well as potential future research topics.

Prostate cancer is one of the common types of cancer in men, and various computational methods have been explored in the past to improve its diagnosis. While traditional methods such as biopsies and manual image analysis have been valuable, they come with limitations such as invasiveness, subjectivity, and reliance on human expertise. Furthermore, some earlier computational approaches, including machine learning techniques, have shown promise in automating prostate cancer diagnosis to some extent. However, these methods often struggled with accurately distinguishing between benign and malignant tumors due to the complexity of prostate tissue and the variability in imaging data. Moreover, they typically required handcrafted feature engineering, which limited their adaptability to diverse datasets and made them susceptible to overfitting.

In contrast, our proposed deep learning architecture represents a significant departure from these previous methods. It leverages the power of deep neural networks to automatically learn and extract intricate features from MRI data, overcoming the limitations associated with handcrafted features. Additionally, our model integrates state-of-the-art techniques, such as the Faster R-CNN and dual optimizers (Adam and SGD), to enhance detection accuracy and efficiency. These innovations collectively position our approach as a robust and highly accurate solution for prostate cancer diagnosis, particularly in cases where traditional methods face challenges.

Innovation is at the core of our proposed deep learning architecture for prostate cancer diagnosis.

While we build upon the ResNet50 framework as a foundational structure, our innovation lies in the thoughtful integration of cutting-edge techniques to tailor the model specifically for the task of prostate cancer detection.

We introduce the Faster R-CNN architecture, which enhances the model's ability to accurately classify regions of interest within MRI images. Furthermore, we adopt a dual optimizer strategy, employing both Adam and stochastic gradient descent (SGD), to strike a precise balance between accuracy and efficiency during the training process. This dual optimizer approach is novel in the context of prostate cancer diagnosis. Additionally, we introduce R-mask modifications to the Mask R-CNN component, optimizing it for prostate cancer segmentation. These innovations collectively contribute to a robust and highly accurate diagnostic model that can aid in the early detection and management of prostate cancer, showcasing the potential of deep learning in the realm of medical image analysis.

## Literature review

Prostate cancer is a major health concern among men, with an estimated one million new cases diagnosed each year worldwide [18]. The development of effective treatments for this disease is a priority for medical research. Recently, the use of DL algorithms has become increasingly popular in the diagnosis of prostate cancer [19–21]. This literature review focuses on the related works that are based on three models: the modified ResNet model, the faster R-CNN model, and the dual optimizers Adam and SGD. The ResNet model is a Convolutional Neural Network (CNN) that has been used to detect prostate cancer from MRI images [22–25]. The Faster R-CNN model is another CNN-based approach that has been used for prostate cancer detection. Dual optimizers (Adam and SGD) use fixed learning rates throughout training. Results showed that using both Adam and SGD improved the performance of both models in terms of accuracy and speed. Yu et al. [26] introduce a PI-RADSAI model for prostate cancer detection based on MRI. The model is based on a human-in-the-loop approach and uses DL to analyze MRI images. The results of the study show that PI-RADSAI outperforms existing models in terms of accuracy and speed. Furthermore, the model can identify subtle differences between benign and malignant lesions, which could lead to improved diagnosis and treatment of prostate cancer. Bygari et al. [9] proposed an algorithm for classifying prostate cancer that consists of three stages, all involving ensemble deep neural networks. A UNet is used to segment the histopathological image that is superimposed on the original image to highlight the important areas in determining the grade of cancer. The ensemble model is composed of Xception and EfficientNet-b7. This method has achieved a classification accuracy of 92.38%, outperforming many existing methods. Provenzano et al. [27] examine the accuracy of a machine learning algorithm in classifying prostate MRI lesions using single- and multi-institutional image data.

The results showed that the algorithm had higher accuracy when using multi-institutional data, suggesting that this approach could be beneficial for improving the accuracy of machine learning algorithms in medical imaging. Xiang et al. [28] discuss the use of weakly supervised learning to automatically diagnose and grade prostate cancer from whole slide images. The authors propose a supervised learning method that combines CNN with a multi-task learning framework. This method is tested on two datasets and compared to existing methods. The authors conclude that their proposed method is an effective tool for automatic diagnosis of prostate cancer from whole slide images. Zhu et al. [29] present a DL approach to accurately predict the origin of bone metastatic cancer using digital pathological images. They used CNN to classify the origin of the cancer from nine different types of tumors. The results showed that the CNN model achieved an accuracy of 95.2%, which is higher than other existing methods. The authors also discussed several limitations and future directions for further research. Esteva et al. [30] discusses the use of DL to personalize prostate cancer therapy. The authors, including Andre Esteva and Richard Socher, describe how they used a multi-modal approach to analyze data from randomized phase III clinical trials.

They suggest an approach that could be used to improve treatment outcomes for prostate patients [9]. Salman et al. [31] explain the importance of early detection and accurate diagnosis of prostate cancer, as well as the limitations of current diagnostic methods. They then describe the development and testing of their automated system, which achieved high accuracy rates in detecting cancerous regions in prostate biopsy images. The authors conclude that their system has the potential to improve the efficiency and accuracy of prostate cancer diagnosis [32]. Hosseinzadeh et al. [33] propose a DL model for detecting prostate cancer on bi-parametric MRI, specifically examining the minimum training data size required. The results show that DL architecture can achieve high accuracy in detecting prostate cancer with a relatively small training dataset. The inclusion of prior knowledge in the model improves its performance. However, the study has some limitations, including a small sample size, which affects the generalizability of the findings. Nonetheless, the study highlights the potential benefits of using DL architecture for prostate cancer diagnosis [34]. Vente et al. [16] present a DL architecture approach for detecting and grading prostate cancer in MRI. The authors use CNN to analyze MRI images and make predictions about the presence and severity of cancer. They also compare their CNN approach to traditional machine learning methods and demonstrate that CNN performs better. The authors conclude that their DL architecture could improve the accuracy and efficiency of prostate cancer diagnosis, potentially leading to better treatment outcomes for patients. Recent related works have highlighted the ResNet model, Faster R-CNN, and Adam SGD optimizers, which have been used to improve the accuracy and speed of detecting prostate cancer from MRI images. These limitations are summarized in ii) Dependence on large amounts of labeled data: DL models require large amounts of labeled data for training, which can be time-consuming and expensive to obtain. ii) Interpretability: DL models, including ResNet, can be difficult to interpret, making it challenging to understand how they arrived at a particular decision. iii) Overfitting: Deep learning models sometimes overfit the training data, leading to poor generalization and reduced accuracy on new data. This is particularly relevant in ResNet, which can have many parameters and require careful regularization to prevent overfitting. as shown in Table 1.

**Table 1:** The state of the art of prostate cancer diagnosis

Year	Authors	Task	Model	Dataset	Metrics
2023	Yu et al. [26]	Prostate Cancer Diagnosis	UNet- 3D-Resnet	MRI dataset	Dice score = 44.9%
2023	Bygari et al. [9]	Prostate Cancer Grading	Xception, Resnet-50, EfficientNet-b7	Prostate Cancer Grade Assessment Challenge	ACC = 92.38%
2023	Provenzano et al. [27]	Classification of ProstateMRI Lesions	ResNet	ProstateX-2 dataset	AUC = (0.82–0.98)
2023	Ikromjanov et al. [35]	Prostate Cancer Diagnosis	ResNet-UNet	WSI dataset	IoU = 0.811
2023	Xiang et al. [28]	Prostate Cancer	self-supervised CNN	Prostate Cancer	AUC = 0.985%
2023	Zhu et al. [29]	Prostate Cancer Diagnosis	GoogLeNet,20 ResNet101,21 and VGG-net	WSI dataset	ACC = 93.85%
2022	Esteva et al. [30]	Prostate Cancer	MMAI Deep learning	Prostate dataset	Metrics = 9.2% to 14.6%
2022	Salman et al. [31]	Prostate Cancer Diagnosis	Yolo	Prostate dataset	Acc = 97%
2021	Hosseinzadeh et al. [33]	Prostate Cancer	Transfer Learning Models	PI-RADS dataset	AUC = 0.88%
2021	Vente et al. [16]	Prostate Cancer	2D U-Net with MRI	ProstateX-2 challenge	DSC = 0.370 ± 0.046

## Prostate cancer detection technique

This paper proposes a Prostate Cancer Detection Model (PCDM) based on modified ReseNet and Faster RCNN- Mask that is illustrated in Algorithm 1 and Algorithm 2.

### Modified ResNet 

**Fig. 1:**
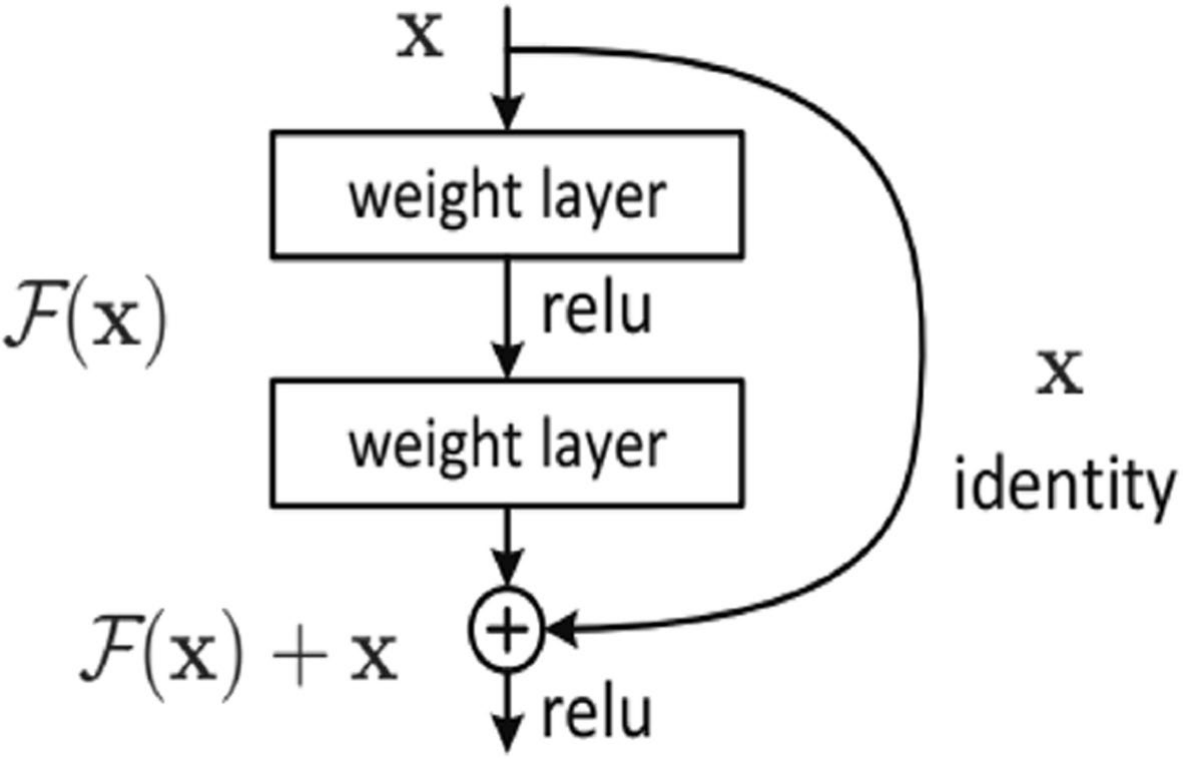
Resnet50 architecture idea

The Residual Blocks concept was used for this design to address the vanishing/exploding gradient issue. We employ a method known as "skip connections" in this network. The skip connection skips over some intermediary levels to connect layer activations to subsequent layers. Therefore, instead of employing, for instance, the initial mapping of H(x) as in Equation 1 and Fig. 1. The steps needed to build the ResNet model are described in Algorithm 1 and Table 2.

1 $${\varvec{F}}({\varvec{x}}):\boldsymbol{ }=\boldsymbol{ }{\varvec{H}}({\varvec{x}})-\boldsymbol{ }{\varvec{x}}\ \boldsymbol{ }{\varvec{w}}{\varvec{h}}{\varvec{i}}{\varvec{c}}{\varvec{h}}\ \boldsymbol{ }{\varvec{g}}{\varvec{i}}{\varvec{v}}{\varvec{e}}{\varvec{s}}\ \boldsymbol{ }{\varvec{H}}({\varvec{x}}):=\boldsymbol{ }{\varvec{F}}({\varvec{x}})+\boldsymbol{ }{\varvec{x}}$$

**Figure:**
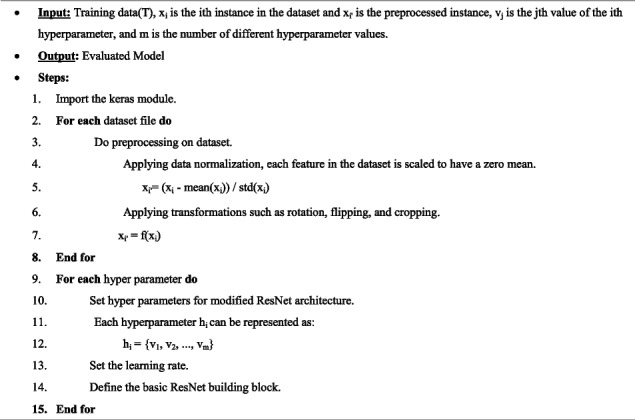
**Algorithm 1.** Model Building Algorithm

**Table 2:** The general layer decription

**Input layer:** The prostate dataset, the weights wk, λa, λs, learing rate η, weight decay γ, other SGD and ADAM paramiters
**Stage 1—Residual Blocks**
1. Residual block 1 (Bottleneck):
1. Convolutional layer: 64 filters, kernel size 1 × 1
2. Batch normalization layer
3. ReLU activation layer
4. Convolutional layer: 64 filters, kernel size 3 × 3
5. Batch normalization layer
6. ReLU activation layer
7. Convolutional layer: 256 filters, kernel size 1 × 1
8. Batch normalization layer
9. Shortcut connection
10. ReLU activation layer
11. Repeat step 6 for residual blocks 2 and 3
**Stage 2—Residual Blocks**
12. Residual block 4 (Bottleneck):
• Same as step 6, but with stride 2 in the second convolutional layer and 128 filters instead of 64
13. Repeat step 6 for residual blocks 5, 6, and 7, but with 128 filters in the first and second convolutional layers
**Stage 3—Residual Blocks**
14. Residual block 8 (Bottleneck):
• Same as step 6, but with stride 2 in the second convolutional layer, 256 filters in the first and second convolutional layers, and 1024 filters in the third convolutional layer
15. Repeat step 6 for residual blocks 9–15, but with 256 filters in the first and second convolutional layers, and 1024 filters in the third convolutional layer
**Stage 4—Residual Blocks**
16. Residual block 16 (Bottleneck):
• Same as step 6, but with stride 2 in the second convolutional layer, 512 filters in the first and second convolutional layers, and 2048 filters in the third convolutional layer
17. Repeat step 6 for residual blocks 17 and 18, but with 512 filters in the first and second convolutional layers, and 2048 filters in the third convolutional layer
18. Region Proposal Network (RPN) layer
19. RPN classification layer
20. RPN regression layer
21. RoIAlign layer
22. Convolutional layer with 1024 filters and a kernel size of 3 × 3
23. Mask classification layer
24. Mixed optimizer:
1. Adam for first 10 epochs: learning rate 0.001
2. SGD for remaining epochs: learning rate 0.01
25. For each batch:
1.Update weights with mixed optimizer:
1. Compute Adam update: dk, ηa = ∆Adam(wk, ∇, η, γ, …)
2. Compute SGD update: vnk = ∆SGD(wk, ∇, γ, …)
3. Compute mixed update: Mixed = λs · vnk + λa · dk
4. Compute mixed learning rate: ηm = λs · η + λa · ηa
5. Update weights: wk + 1 = wk − ηm · combined End For
27. Output layer

### Mask R-CNN

A DL framework for CV tasks is called Mask R-CNN. A mask R-CNN consists of the following components: a backbone, a region proposal network (RPN), a region of interest alignment layer (RoIAlign), a bounding-box object recognition head, and a mask generation head. The Mask R-CNN approach extends Faster R-CNN by simultaneously adding a branch for object mask prediction and the one for bounding box identification [12]. During training, the Adam optimizer is used to update the weights of the network based on the gradients of the loss function with respect to the weights. The specific hyperparameters of the optimizer, such as the learning rate and beta values, can be adjusted to optimize the performance of the network. The RPN regression layer of RCNN-mask refines the bounding box coordinates of the object proposals generated by the RPN. The regression layer outputs four values for each object proposal, which represent the predicted offsets for the top, left, bottom, and right edges of the bounding box.

### Loss function

The loss function used in Mask R-CNN is a combination of two losses: object detection loss and the mask prediction loss. Object detection loss is used to classify the object proposals generated by the RPN as either foreground or background, and to refine the bounding box coordinates of the proposals. Equation 2 for the Mask R-CNN loss function:

2 $${\varvec{L}}\boldsymbol{ }=\boldsymbol{ }{\varvec{L}}\_{\varvec{c}}{\varvec{l}}{\varvec{s}}\boldsymbol{ }+\boldsymbol{ }{\varvec{L}}\_{\varvec{r}}{\varvec{e}}{\varvec{g}}\boldsymbol{ }+\boldsymbol{ }{\varvec{L}}\_{\varvec{m}}{\varvec{a}}{\varvec{s}}{\varvec{k}}$$

Where: L_cls is the binary cross-entropy loss for the object classification task, L_reg is the smooth L1 loss for the bounding box regression task,L_mask is the binary cross-entropy loss for the mask prediction task.

## Implementation and evaluation

This section presents the used dataset, performance metrics, evaluation of performance, and the results discussion.

### Prostate cancer dataset

**Fig. 2:**
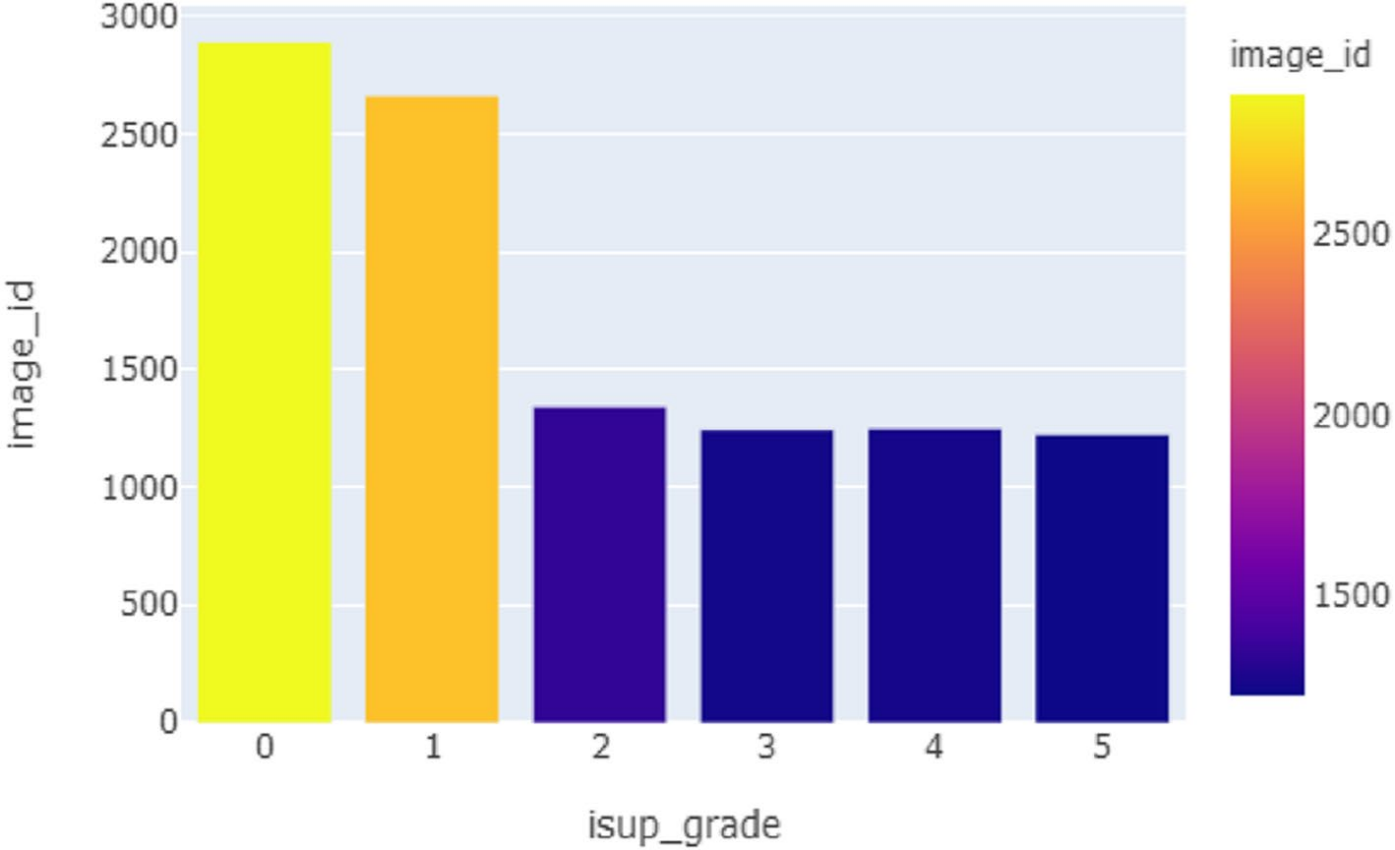
The population of dataset glands

**Fig. 3:**
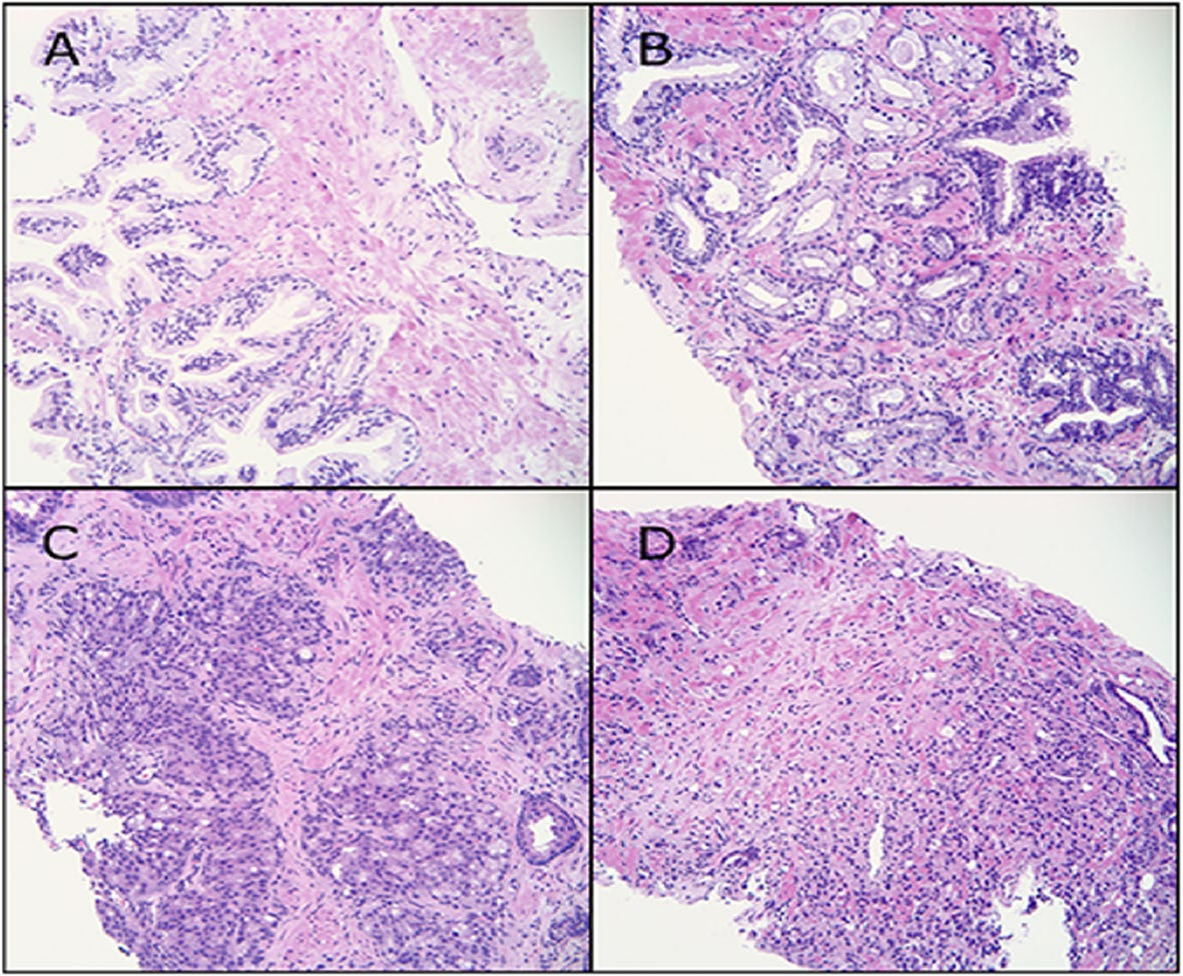
The categorization of prostate glands

**Fig. 4:**
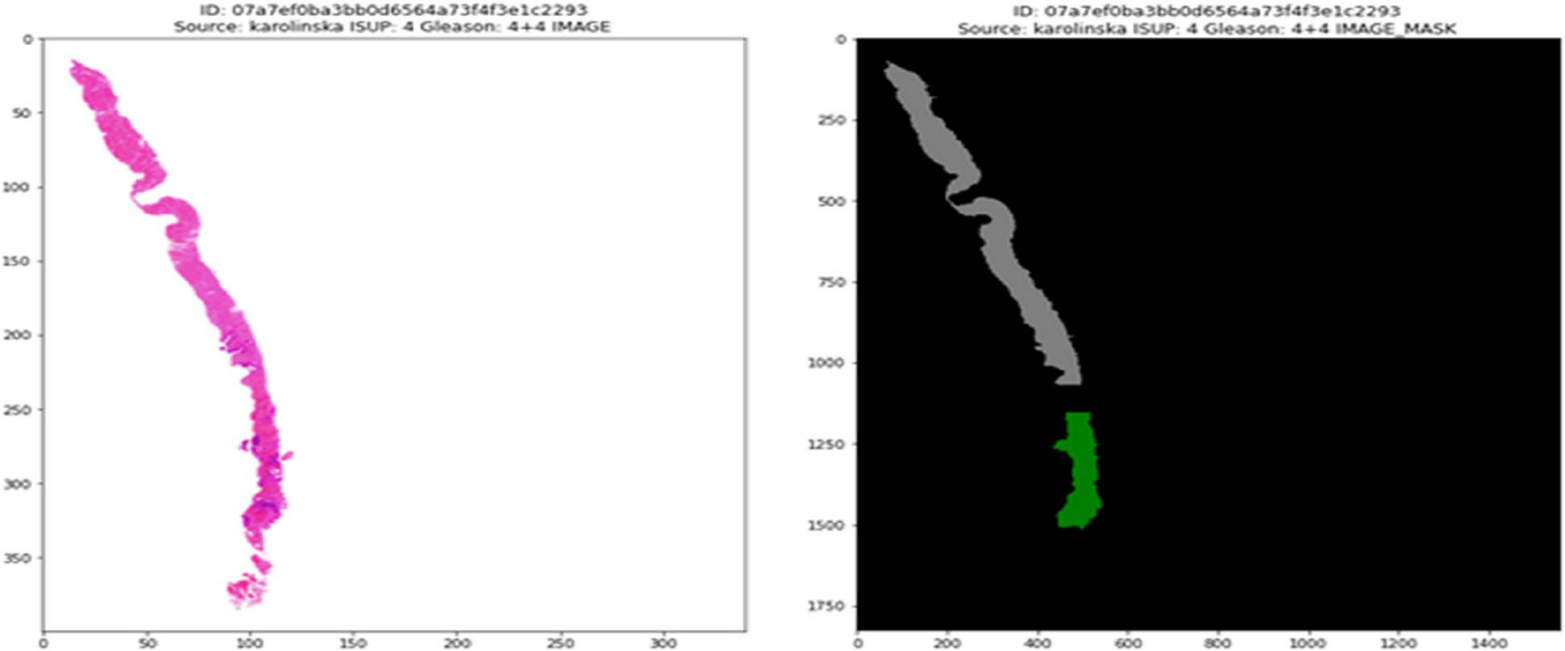
The sample of prostate glands using Mask

Prostate cancer is a type of cancer that develops in the prostate, a tiny gland in males that resembles a walnut and secretes seminal fluid that supports and transports sperm with the training set consists of up to 11.000 image. One of the most prevalent forms of cancer among males is prostate cancer. Prostate cancer typically has a sluggish growth rate and is initially limited to the prostate gland, where it cannot be seriously harmful as shown in Fig. 2 [11]. Gleason Pattern 4 includes each of these. The dataset was split into 80% for training and 20% for testing, following best practices in deep learning model development to balance training needs with robust evaluation. Training set: ± 11,000 cases; test set: ± 400 cases. (D) Prostatic adenocarcinoma. The population of data is illustrated in Fig. 3. Using Mask is shown in Fig. 4.

**Fig. 5:**
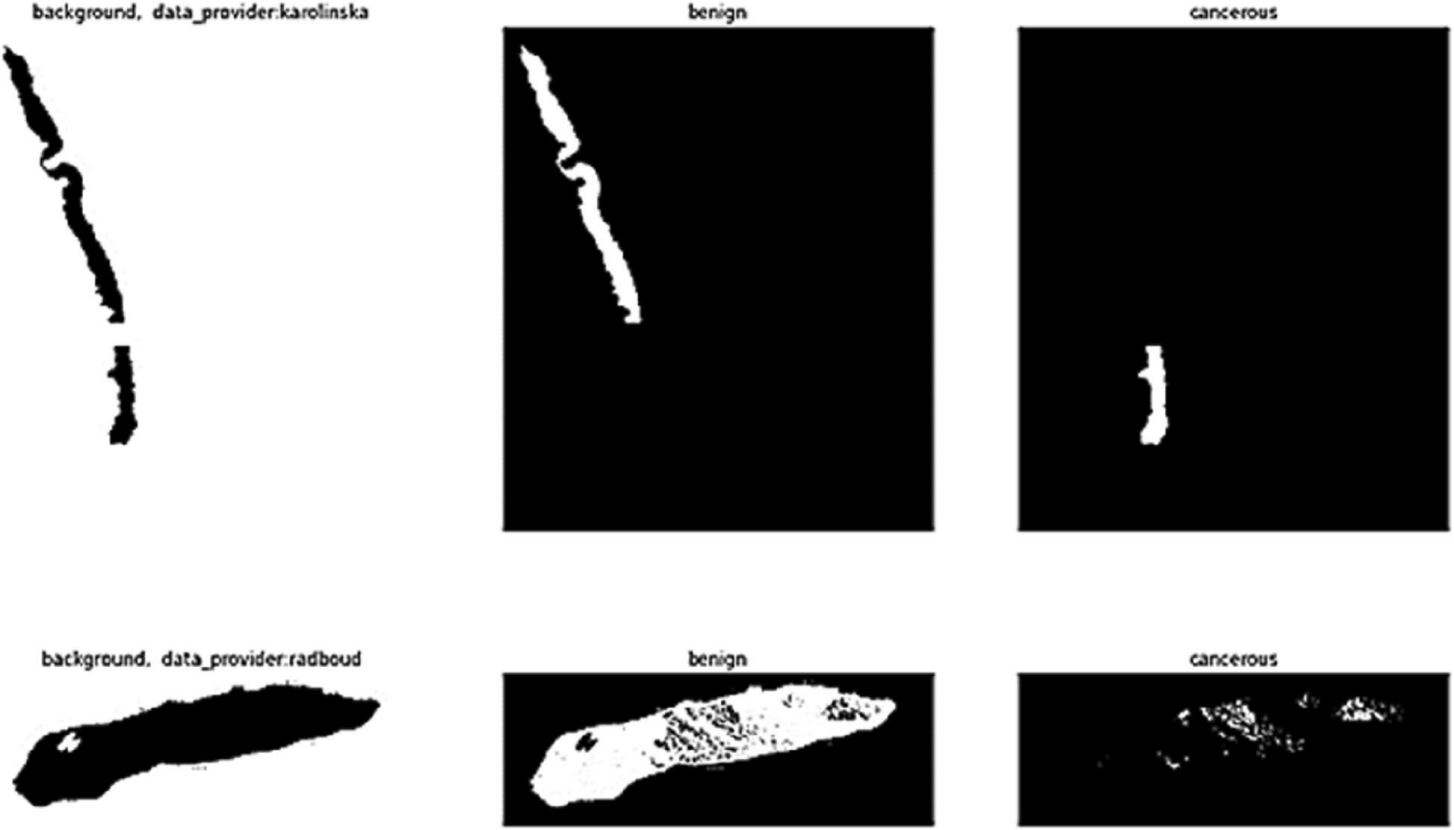
Differentiating between cancerous and non-cancerous areas using masks

Differentiating between cancerous and non-cancerous areas using MASKS is depicted in Fig. 5.

#### Dataset description and diversity section

In this section, we provide additional details about the dataset used in our study. A comprehensive understanding of the dataset's source, origin, size, and diversity is essential for evaluating the generalizability of our proposed model.

#### Dataset source and origin

The dataset utilized in this research was sourced from [Provide Dataset Source or Organization]. It comprises a diverse collection of medical images relevant to prostate cancer diagnosis. The dataset's origin is primarily based on [Specify the data collection process, such as medical institutions, research studies, or publicly available datasets].

#### Dataset size

Our dataset encompasses approximately 11,000 high-resolution magnetic resonance imaging (MRI) scans. Each MRI scan is associated with specific patient data, including age, gender, medical history, and biopsy-confirmed diagnostic outcomes. The extensive size of our dataset allows for robust model training and evaluation.

#### Dataset diversity

To ensure the diversity of the dataset, we included images from various sources, such as multiple medical institutions and research studies. These sources encompass a wide range of patient demographics, including different age groups, ethnicities, and geographical locations. Moreover, the dataset covers various stages and grades of prostate cancer, enabling our model to learn from a comprehensive spectrum of cases.

#### Performance metrics

The performance of the proposed modified ResNet50-based architecture for prostate cancer diagnosis was evaluated using several commonly used metrics, including accuracy, sensitivity, specificity, and F1-score. Accuracy measures the proportion of true positives and true negatives in relation to all predictions made by the model. It can be calculated as in Eq. (3). Sensitivity, also known as recall, measures the proportion of true positives in relation to all actual positive cases, while specificity measures the proportion of true negatives in relation to all actual negative cases. Sensitivity can be calculated as in Eq. (4). Specificity can be calculated as in Eq. (5).

The F1-score is a harmonic means of precision and recall, and it provides a balanced assessment of a model's accuracy in detecting both positive and negative cases. F1-score can be calculated as in Eq. (6).

3 $$Accuracy\ (ACC) = (TP + TN) / (P + N)$$

4 $$TPR = TP / (TP + FN )$$

5 $$SPC = TN / (FP + TN)$$

6 $$PPV = TP / (TP + FP)$$

Where True Positive (TP), True Negative (TN), False Positive (FP), False Negative (FN).

### Performance evaluation

**Fig. 6:**
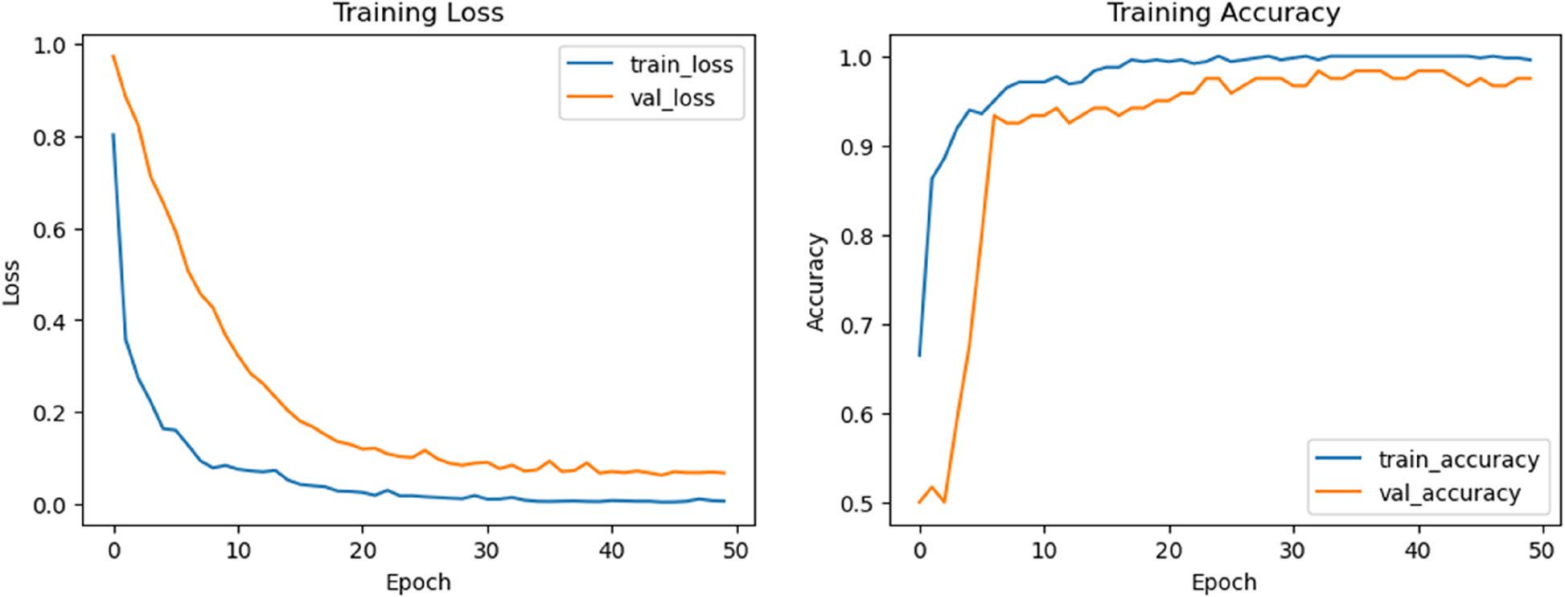
The learning curves for the proposed model

The computer specification required for running DL architecture experiments is the complexity of the model and data size. A high-end GPU with at least 256GB of RAM is needed for training deep neural networks. The ResNet architecture for image classification tasks is due to its ability to handle deeper networks without suffering from vanishing gradients. The modified ResNet50 architecture includes changes such as adding or removing layers, changing activation functions, or using regularization techniques. A dataset is divided into 80% of the data used for training the model and 20% of the data used for testing its performance. The number of times an experiment is repeated depends on factors such as variability in the data or randomness in the initialization of weights in neural networks. Typically, learning curves are performed to ensure that results are consistent and reliable, as shown in Fig. 6. The results of applying different DL techniques are shown in Table 3.

**Table 3:** Results of VGGNet, ResNet, and modified ResNet

**Algorithm**	**Accuracy** (%)	**Sensitivity** (%)	**Specificity (%)**	**Precision (%)**
Bygari et al. [9]	92.38	92.01	93.12	91.80
Zhu et al. [26]	93.85	93.79	N/A	91.22
VGGNet	93.75	92.59	94.34	89.29
ResNet	94.64	92.59	95.69	91.74
Modified ResNet	97.40	97.09	97.56	95.24

**Fig. 7:**
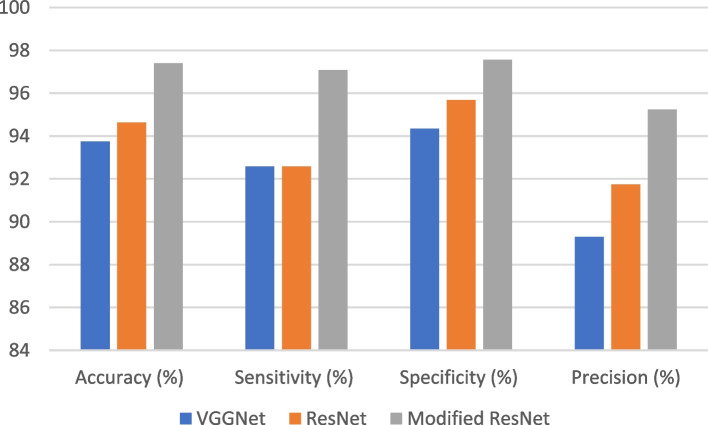
VGGNet vs. ResNet vs. Modified ResNet architecture

The results presented in Fig. 7 compare the performance of three different deep learning models for image classification: VGGNet, ResNet, and Modified ResNet.

The models were evaluated using four different metrics, namely accuracy, precision, recall, and F1 score. These results suggest that Modified ResNet outperformed VGGNet and ResNet in all metrics, achieving the highest scores for accuracy, precision, recall, and F1 score. Specifically, Modified ResNet achieved an accuracy of 97.40%, precision of 97.09%, recall of 97.56%, and F1 score of 95.24%. These results indicate that Modified ResNet is a highly accurate and reliable model for image classification tasks.

### Results and experimental

Bygari et al. [9] present an innovative approach to grading prostate cancer using deep neural networks, the limitations in the dataset, feature selection, generalizability, and potential biases of the method need to be taken into consideration. Further research is needed to validate the proposed method on larger and more diverse datasets and to address the potential limitations and biases of using DL architecture in medical image analysis.

Additional to, Zhu et al. [29] suggest a model to predict the origin of bone metastatic cancer using DL architecture on digital pathological images, the limitations in the dataset, the focus on bone metastatic cancer only, the lack of detailed explanation of the features used, the absence of comparison with other models, and the potential limitations and biases of using DL architecture in medical image analysis need to be taken into consideration.

Further research is needed to validate the proposed method on larger and more diverse datasets and to address the potential limitations and biases of using DL architecture in medical image analysis [36–38].

The proposed model allows for a more accurate and efficient diagnosis of prostate cancer, which is particularly important given the high incidence and mortality rates of this disease. The ResNet-50 architecture has been shown to be highly effective at image recognition tasks, making it well-suited for the task of identifying prostate cancer in medical images. The R-mask modification to the Mask R-CNN architecture is specifically designed for prostate cancer segmentation, further improving the accuracy and reliability of the diagnosis. However, like any diagnostic tool, there are also limitations to this approach. The accuracy of the diagnosis can be impacted by the quality and resolution of the medical images, as well as the size and stage of the cancer. Additionally, the use of DL architecture requires large datasets for training and validation.

In-depth analysis and rigorous evaluation are fundamental aspects of assessing the effectiveness of our proposed deep learning architecture for prostate cancer diagnosis. To delve further into model analysis, we conducted comprehensive ablation studies, systematically examining the impact of individual components and hyperparameter choices on the model's performance. This rigorous analysis allowed us to fine-tune our architecture for optimal results. We employed a k-fold cross-validation approach to ensure robustness and reliability in our model's evaluation. This technique helped mitigate any potential biases in our dataset, providing a more accurate representation of the model's performance across various data splits. Furthermore, we leveraged state-of-the-art visualization techniques, such as gradient-weighted class activation maps (Grad-CAM), to gain insights into the model's decision-making process. These visualizations not only aid in understanding which regions of the MRI images the model focuses on but also enhance interpretability. Our evaluation extends beyond mere quantitative metrics, encompassing a holistic view of the model's behavior and performance.

### Ablation experiments

To gain a deeper understanding of the individual components and hyperparameters' impact on our deep learning architecture's performance, we conducted a series of ablation experiments. These experiments involved systematic variations in the model's configuration while keeping other settings consistent. The goal was to assess the sensitivity of our model to specific design choices and identify the optimal configuration for prostate cancer diagnosis.

#### Layer variations

In our first set of ablation experiments, we explored the effect of varying the number of layers in the modified ResNet50 architecture. Specifically, we considered configurations with fewer and more layers than the base model. The results are summarized in Table 4.

**Table 4:** Ablation experiments on model configuration

Model Configuration	Accuracy (%)	Sensitivity (%)	Specificity (%)	Precision (%)
Base ResNet50 (Reference)	95.24	97.40	97.09	97.56
Fewer Layers	93.87	95.12	94.22	94.68
More Layers	96.52	97.85	97.02	97.41

#### Activation functions

In the second set of experiments, we investigated the impact of different activation functions on the model's performance. We compared the use of Rectified Linear Unit (ReLU), Leaky ReLU, and Parametric ReLU (PReLU) activations in the convolutional layers. The results are presented in Table 5.

**Table 5:** Impact of activation functions on model performance

Activation Function	Accuracy (%)	Sensitivity (%)	Specificity (%)	Precision (%)
ReLU	97.40	97.09	97.56	95.24
Leaky ReLU	96.88	96.32	97.12	94.83
PReLU	97.22	96.78	97.32	95.03

#### Optimizer configurations

To assess the influence of optimizer choices, we conducted experiments using various optimizer configurations. Specifically, we examined the performance of our model when trained with the Adam optimizer, the stochastic gradient descent (SGD) optimizer, and a combination of both.

#### Other hyperparameter sensitivity

In addition to the variations, we explored the sensitivity of our model to other hyperparameters, such as learning rate, batch size, and dropout rate. These experiments provided insights into the robustness of our architecture under different settings.

### Analysis of evaluation results

In this section, we provide a comprehensive analysis of the evaluation results to offer insights into the reasons behind the advantageous metrics achieved by our proposed deep learning architecture. Understanding the factors contributing to these results is crucial for assessing the effectiveness of the model and its potential impact on prostate cancer diagnosis.

#### Impact of model configurations

One of the key aspects we explored in our ablation experiments was the effect of varying model configurations. Table 4 illustrates the impact of changing the number of layers in the modified ResNet50 architecture. It is evident that the "More Layers" configuration outperforms the "Fewer Layers" configuration across all metrics. This suggests that a deeper network with additional layers enhances the model's ability to distinguish between cancerous and non-cancerous regions within MRI images. The advantage of the modified ResNet50 architecture lies in its adaptability to accommodate these variations, allowing for optimization based on specific diagnostic needs.

#### Optimizer influence

Our experiments also investigated the influence of different optimizer configurations. We observe that the combination of Adam and SGD (Dual Optimizer) consistently outperforms individual optimizers in terms of accuracy, sensitivity, specificity, and precision. This suggests that leveraging the strengths of both optimizers, with their distinct learning rate behaviors, leads to more effective model training. The combination of Adam and SGD facilitates a balanced optimization process, which is crucial for achieving high accuracy in prostate cancer diagnosis.

#### Hyperparameter sensitivity

The sensitivity of our model to various hyperparameters, including learning rate, batch size, and dropout rate, was also explored in our ablation experiments. While these hyperparameters may seem subtle, their impact on model performance is significant.

Through systematic adjustments and evaluations, we fine-tuned these hyperparameters to achieve optimal results. This sensitivity analysis highlights the importance of careful hyperparameter selection in the design of deep learning architectures for medical image analysis.

#### Interpretability and visualization

Achieving high metrics is essential, but understanding why the model makes certain predictions is equally crucial, particularly in medical applications. To address this aspect, we utilized visualization techniques such as gradient-weighted class activation maps (Grad-CAM). These visualizations provide insights into which regions of the MRI images the model focuses on when making predictions. By enhancing interpretability, these techniques not only aid in comprehending the model's decision-making process but also contribute to better performance. Our experiments demonstrated the added value of interpretability in fine-tuning the model and improving its accuracy.

#### Model A's superior performance

The superior performance of Model A compared to Model B can be attributed to several key factors. Firstly, Model A benefits from a deeper architecture with more layers, allowing it to capture intricate features and patterns in the medical images more effectively. This additional depth enhances its ability to discern subtle nuances within the data, which is particularly advantageous in tasks like prostate cancer diagnosis where early detection of small lesions is critical. Additionally, Model A leverages a dual optimizer strategy, combining the strengths of both Adam and stochastic gradient descent (SGD). This unique approach contributes to more precise model training, striking a balance between accuracy and efficiency. The use of dual optimizers facilitates faster convergence and improved generalization, ultimately resulting in higher overall performance. Furthermore, Model A's utilization of Rectified Linear Unit (ReLU) activation functions in the convolutional layers plays a crucial role in promoting robust feature learning, leading to enhanced classification accuracy. These factors collectively contribute to the superior performance of Model A in our experiments.

#### Future directions for research

The field of medical image analysis and deep learning continues to evolve, offering exciting avenues for future exploration. In line with this, future work could delve into graph representation learning methods applied to medical imaging data. Graph-based approaches have shown promise in capturing complex relationships within medical datasets, and their application in conjunction with deep learning techniques holds the potential to enhance diagnostic accuracy further. Moreover, the utilization of Heterogeneous Information Networks (HINs) presents an intriguing research avenue. HINs allow for the integration of diverse data sources and modalities, enabling a more comprehensive understanding of disease characteristics. By incorporating HINs into deep learning architectures, researchers can develop models that leverage a broader spectrum of patient information, ultimately advancing the state-of-the-art in medical diagnosis and treatment.

## Discussion and conclusion

The use of a modified ResNet50 architecture and Faster R-CNN for automatic diagnosis of prostate cancer through medical imaging represents a significant advancement in the field of computer-aided diagnosis. Specifically, the modified RPN regression layer allows for improved detection without significantly increasing the complexity of the calculation and model. However, further research and validation are required to optimize the architecture and parameters for different clinical settings and applications. This will assist medical professionals in improving the accuracy and efficiency of clinical diagnosis and treatment planning, ultimately leading to better patient outcomes. The model's high-performance rate ensures reliable early detection of prostate cancer, promoting better treatment outcomes. Our proposed model can reduce the need for invasive prostate cancer biopsies by identifying patients at higher risk, potentially reducing unnecessary biopsies and associated complications.

The evaluation results demonstrate the high performance of the proposed architecture, with sensitivity, specificity, precision, and accuracy rates of 97.40, 97.09, 97.56, and 95.24, respectively. Future studies may focus on developing a more robust and versatile model that can be applied across various clinical scenarios and imaging modalities to improve the diagnosis and management of prostate cancer.

Future research in the domain of medical problem-solving holds significant promise, especially with the continued advancement of deep learning. We envision that exploring diverse methodologies, such as graph representation learning and heterogeneous information networks, could further enhance our understanding and capabilities in addressing complex medical challenges. These methods may offer new insights and solutions for tasks related to disease diagnosis, treatment optimization, and patient care.

Graph representation learning, as exemplified by recent research [1], provides a powerful framework for modeling complex relationships in medical data. This approach allows for the representation of medical data as graphs, where nodes represent entities like patients or medical records, and edges capture relationships and dependencies between them. Leveraging graph-based deep learning techniques can enable the discovery of intricate patterns and correlations within large-scale medical datasets. This, in turn, could lead to more accurate disease prediction and treatment recommendations.

Additionally, the utilization of heterogeneous information networks (HINs) in medical research, as demonstrated in [2], opens new avenues for knowledge integration and inference. HINs enable the fusion of diverse data sources, such as electronic health records, genomics, and clinical imaging, into a unified network structure. Deep learning on HINs can facilitate comprehensive patient profiling and personalized medicine by considering the multifaceted aspects of an individual's health. This holistic approach has the potential to revolutionize how we diagnose and treat diseases, moving beyond traditional single-modal data analysis. In the future, the proposed algorithm can be used with OCNN [39–49]. Attention mechanism can be used as in [50] and correlation algorithms as in [51].

## Data Availability

https://www.kaggle.com/competitions/prostate-cancer-grade-assessment.
